# Radiomics and artificial intelligence in prostate cancer: new tools for molecular hybrid imaging and theragnostics

**DOI:** 10.1186/s41747-022-00282-0

**Published:** 2022-06-15

**Authors:** Virginia Liberini, Riccardo Laudicella, Michele Balma, Daniele G. Nicolotti, Ambra Buschiazzo, Serena Grimaldi, Leda Lorenzon, Andrea Bianchi, Simona Peano, Tommaso Vincenzo Bartolotta, Mohsen Farsad, Sergio Baldari, Irene A. Burger, Martin W. Huellner, Alberto Papaleo, Désirée Deandreis

**Affiliations:** 1 Medical Physiopathology — A.O.U. Città della Salute e della Scienza di Torino, Division of Nuclear Medicine, Department of Medical Science, University of Torino, 10126 Torino, Italy; 2 Nuclear Medicine Department, S. Croce e Carle Hospital, 12100 Cuneo, Italy; 3 Department of Nuclear Medicine, University Hospital Zurich, University of Zurich, 8006 Zurich, Switzerland; 4 Nuclear Medicine Unit, Department of Biomedical and Dental Sciences and of Morpho-Functional Imaging, University of Messina, 98125 Messina, Italy; 5 Nuclear Medicine Unit, Fondazione Istituto G. Giglio, Ct.da Pietrapollastra Pisciotto, Cefalù, Palermo, Italy; 6 Medical Physics Department, Central Bolzano Hospital, 39100 Bolzano, Italy; 7 Department of Radiology, Fondazione Istituto G. Giglio, Ct.da Pietrapollastra, Cefalù, Palermo, Italy; 8 Nuclear Medicine, Central Hospital Bolzano, 39100 Bolzano, Italy; 9 Department of Nuclear Medicine, Kantonsspital Baden, 5004 Baden, Switzerland

**Keywords:** Prostate cancer, Positron emission tomography, Artificial intelligence, Radiomics, Theragnostics

## Abstract

In prostate cancer (PCa), the use of new radiopharmaceuticals has improved the accuracy of diagnosis and staging, refined surveillance strategies, and introduced specific and personalized radioreceptor therapies. Nuclear medicine, therefore, holds great promise for improving the quality of life of PCa patients, through managing and processing a vast amount of molecular imaging data and beyond, using a multi-omics approach and improving patients’ risk-stratification for tailored medicine. Artificial intelligence (AI) and radiomics may allow clinicians to improve the overall efficiency and accuracy of using these “big data” in both the diagnostic and theragnostic field: from technical aspects (such as semi-automatization of tumor segmentation, image reconstruction, and interpretation) to clinical outcomes, improving a deeper understanding of the molecular environment of PCa, refining personalized treatment strategies, and increasing the ability to predict the outcome. This systematic review aims to describe the current literature on AI and radiomics applied to molecular imaging of prostate cancer.

## Key points

- Artificial intelligence (AI) and radiomic applied to nuclear medicine provide great help in prostate cancer.
- AI-based methods can improve the magnetic resonance imaging-based attenuation correction.
- AI-based fully automatic tissue segmentation is reaching high accuracy for metastasis detection.
- Radiomic features have the potential to predict tumor aggressiveness better than standardized uptake value.
- Neural networks might even be used to simplify dosimetry for theragnostic applications.

## Introduction

### Prostate cancer

Prostate cancer (PCa) is a major health issue, with an estimated nearly 1.4 million new cases and 375,000 deaths worldwide, with incidence rates ranging from 6.3 to 83.4 per 100,000 men. PCa is the fifth leading cause of cancer death among men in 2020 [1, 2].

Several parameters (*i.e.,* androgen-receptor status, gene expression, growth patterns) are responsible for the heterogeneity of PCa and determine the choice between active surveillance for the more indolent disease, curative treatment (prostatectomy or radiotherapy) for localized disease, or systemic therapy for advanced cancer [1, 3].

Nuclear medicine techniques have gained significant relevance for the evaluation and management of PCa in the last 5 years, providing a total-body assessment of tumor burden, thereby discriminating patients with and without oligometastatic disease and patients with extensive disease.

Recently, several nomograms, derived from the combination of clinical and imaging biomarkers, have been developed for diagnostic, prognostic, predictive, or risk stratification purposes [4, 5]. However, their validation for clinical use requires the use of a considerable amount of data [1, 3]. Further opportunities are provided by radiomics and artificial intelligence (AI), potentially enabling clinicians to improve the overall efficiency and accuracy of using a vast amount of data, improving a deeper understanding of the molecular environment of PCa, refining personalized treatment strategies, and increasing the ability to predict the outcome.

This systematic review aims to describe the basic concepts and the current literature on AI and radiomics applied to molecular imaging of PCa.

### Nuclear medicine in prostate cancer

The most well-known radiopharmaceutical in oncology, 2-deoxy-2-[^**18**^F]fluoro-D-glucose ([^**18**^F]FDG), has only limited use for PCa, mainly in advanced disease (dedifferentiated, metastatic, castration-resistant PCa — mCRPC), especially for accurate systemic treatment selection [6, 7]. Historically, the positron emission tomography (PET) radiotracer choline (labeled with [^**18**^F] or [^**11**^C]) played an important role in Pca, mainly for biochemical recurrence (BCR) detection, partly replaced recently by other more specific radiotracers, which will be discussed below. The disease relapse detection rate of choline PET in the BCR setting depends on the PSA level, ranging from 36% for PSA < 1 ng/mL to 73% for PSA > 3 ng/mL [8, 9]. Another interesting PCa radiotracer is [^**18**^F]flurocyclobutane-1-carboxylic acid (FACBC), which improved the assessment of small lesions in the pelvis and prostatic bed compared to choline, owing to its gastrointestinal/hepatic elimination (absent/reduced urinary accumulation) [10]. Currently, the major players in PCa are [^**68**^Ga] or [^**18**^F] labeled radiotracers based on ligands to the prostate-specific membrane antigen (PSMA), which is highly expressed by prostate cancer cells. In a comparative study, [^**68**^Ga]Ga-PSMA-11 showed better results than [^**18**^F]choline in BCR, with a detection rate of 50% *versus* 12% for PSA < 0.5 ng/mL and 69%–86% *versus* 31%–57% for higher PSA values, respectively [11]. Despite some differences [12], similar results were also demonstrated for [^**18**^F]PSMA-1007 for restaging, with a pooled detection rate of 86% for PSA ≥ 0.5 ng/mL and 49% for PSA < 0.5 ng/mL [13]. However, up to 10% of PCa lesions do not express PSMA [14] or have specific features that reduce their detectability by PSMA-based radiotracers [14]. Therefore, alternative radiotracers are under investigation, such as bombesin (BBN) analogs, that reached a detection rate of 71.8% in patients with conventional negative imaging [15]; androgen receptor (AR) imaging with [^**18**^F]FDHT, which might be useful to assess the feasibility and efficacy of AR-directed pharmaceuticals [16]; and [^**18**^F]NaF for the assessment of PCa bone metastases [17]. Finally, an intriguing molecular scenario is the theragnostic approach with radioligand therapy (RLT), mainly represented by [^**177**^Lu]PSMA. This approach showed a high response rate in advanced mCRPC in phase II and phase III trials, with an advantage in terms of bone pain control and overall survival (OS) [18, 19]. RLT may be combined with other oncologic therapies, and such combination therapy may emerge soon. Therefore, it will be important to assess the heterogeneity of PSMA expression within and among patients, to optimally select patients and identify potential mechanisms of tumor resistance [20].

### Radiomics

Radiomics aims to extract a large number of quantitative characteristics (features) from medical images using data-characterization algorithms and bioinformatics approaches. These features, namely radiomic features (RFs), have the potential to uncover disease characteristics that fail to be appreciated by the naked eye, leading to the possibility to quantify specific tumor attributes and phenotypes. RFs can be divided into morphological features, such as compactness and sphericity; first-order features, which describe the distribution of voxel intensities within the specified tumor volume; second-order static features or texture features, which can characterize the spatial interrelationships of intensity between tumor voxels; and higher-order statistical features [21, 22]. Distinctive RFs can help to better describe the biological behavior of the disease in different settings and, consequently, to develop more accurate decision support models by combining medical imaging data (noninvasive and whole-body biomarkers) with other patient characteristics, such as molecular and histopathological tumor characteristics [23].

### Artificial intelligence

The development of algorithms capable of analyzing data and its properties, using dynamic statistical tools, which tend to improve or “learn” as more data is introduced, falls under the definition of machine learning (ML), which is a field of AI. Through the process of “training,” these algorithms improve in using and mapping the observed variables (“features” or “predictors”) to subdivide the data sample into sets of outcome variables (“labels” or “targets”). Based on “labels,” ML can be classified into three broad subsets: supervised, unsupervised, and reinforcement learning [24, 25].

Supervised learning is based on explicit datasets that have been labeled by the operator; in this case, the algorithms measure the difference between the predicted labels and the known labels (called “ground truth”). Linear and logistic regression, support vector machines (SVMs), random forests, and Naive Bayes classification belong to this group of ML techniques [24].

Principal component analysis, k-means clustering, and autoencoders instead belong to unsupervised learning. Here, the algorithm optimally separates samples into different classes based on characteristics of the training data alone, without the operator having first defined labels [26].

In reinforcement learning, a computer (“agent”) learns to perform a task through repeated trial-and-error interactions with a dynamic environment, without being explicitly programmed and without human intervention [26].

**Fig. 1:**
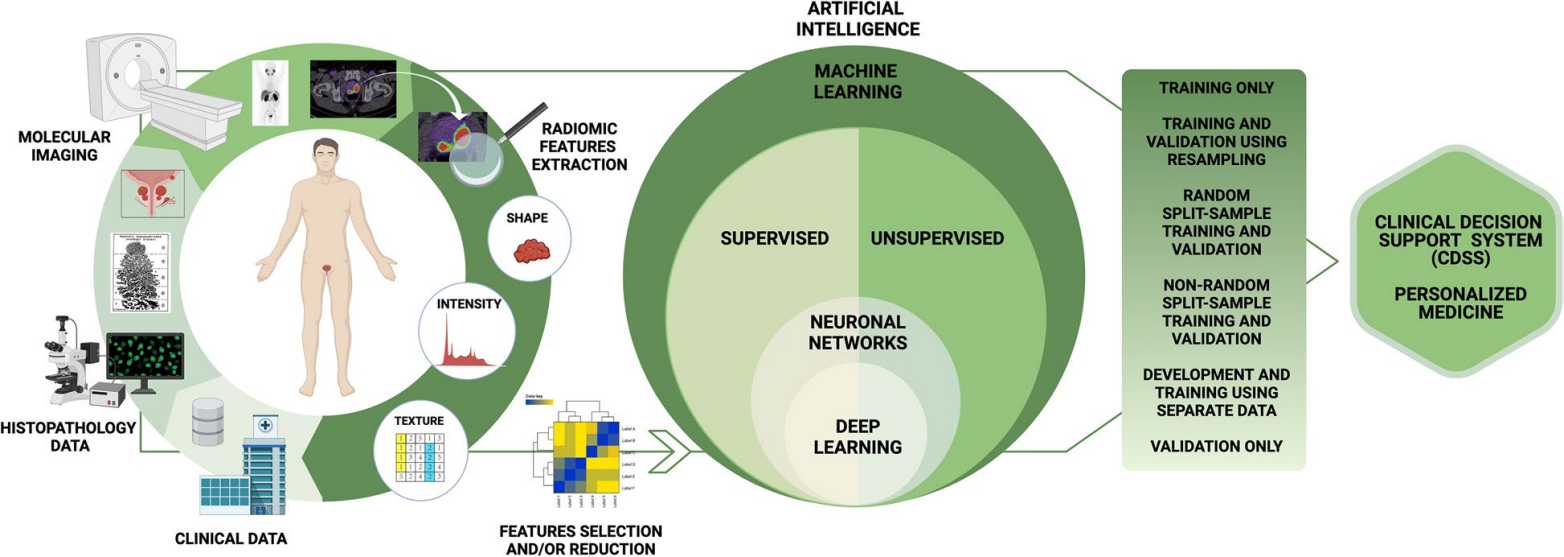
The workflow includes the steps required in a radiomic and artificial intelligence analysis in prostate cancer patients. The first step involves collecting clinical data on patient characteristics, histopathological data on tumor characteristics, and imaging data, with the extraction of radiomic features (such as shape, intensity, and texture features). Radiomic modeling involves three major aspects: feature selection, modeling methodology, and validation. The number of radiomic features that can be extracted from images is virtually unlimited. Once extracted, radiomic features must be selected; redundant or non-robust features against sources of variability must be identified and eliminated through dimensionality reduction techniques, to avoid overfitting problems. The choice of modeling methodology and the identification of optimal machine learning methods for radiomic applications are a crucial step in obtaining robust and clinically relevant results. The choice of a modeling methodology (supervised or unsupervised machine learning method) depends on the setting of the data, the characteristics of the analyzed population, and the experience of the researchers. The model chosen affects prediction and performance in radiomics, and hence, implementations of multiple modeling methodologies are highly desirable. Finally, validation techniques are useful tools for assessing model performance. An externally validated model has more credibility than an internally validated model because data obtained by the first approach are more independent. Validation is essential to verify the repeatability and reproducibility of the model, demonstrating statistical consistency between the training and validation datasets

Based on “features,” ML techniques can be divided into handcrafted (in which the features are explicitly extracted and selected by an operator) and non-handcrafted approaches, in which the process of feature extraction and selection is implicitly incorporated inside the ML algorithm. Among the non-handcrafted approaches, deep learning (DL) is the most widely used. DL can be used on both supervised and unsupervised learning methods. DL approaches are based on artificial neural networks [27], mainly convolutional neural networks (CNNs). Mainly, in the field of medical imaging, CNNs are composed of multiple layers and receive a raw image as initial input (dataset of interest). Then, each layer analyzes and processes the incoming data (input) from the previous layer, sending it to the next layer until an output is extracted from the last layer, which usually identifies a classification label or another evaluable property of the dataset [28]. A resume of the radiomics and AI workflow in PCa is summarized in Fig. 1.

## Material and methods

We searched the PubMed, PMC, Scopus, Google Scholar, Embase, Web of Science, and Cochrane library databases (between January 2010 and November 2021), using the following, both as text and as MeSH terms: “prostate cancer,” “artificial intelligence,” “deep learning,” “machine learning,” “convolutional neural network,” “artificial neural network,” “radiomic,” “segmentation,” “PET,” “PET/CT,” “PET/MR,” “prostate-specific membrane antigen,” “PSMA,” “[^18^F]DCFPyL,” “[^68^Ga]Ga-PSMA-11,” “[^18^F]PSMA-1007,” “[^18^F]flurocyclobutane-1-carboxylic acid,” “FACBC,” “choline,” “gastrin-releasing peptide receptors,” “bombesin,” “[^18^F]NaF,” “bone scintigraphy,” “[^18^F]fluorodeoxyglucose,” “FDG,” “[^68^Ga]RM2,” “177Lu,” “[^177^Lu]PSMA,” “theragnostic,” and “theranostic.”

**Fig. 2:**
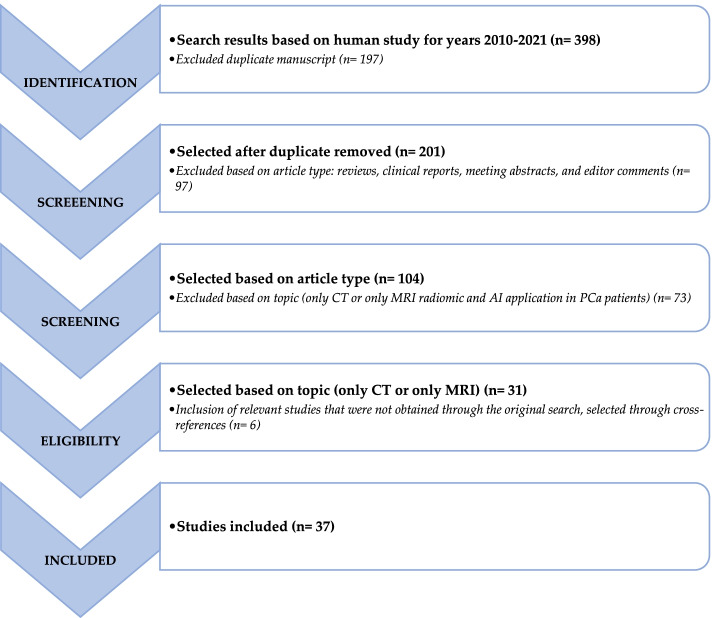
Schematic representation of the performed literature search and the review strategy

No language restriction was applied to the search, but only articles in English were reviewed. The systematic literature search returned 398 articles. According to the Preferred Reporting Items for Systematic Review and Metanalysis (PRISMA) guidelines, after duplicate removal, 37 articles have been considered, fully read, analyzed, and extensively described according to their title and abstract as previously described [29]. We also checked for further relevant articles in the references of the articles included in the retrieved literature. Articles were then grouped into either technical or clinical applications. A graphical representation of the search and review strategy is presented in Fig. 2.

## Technical applications

Several authors have recently attempted to implement PET reconstruction methods through the application of different AI approaches regarding reconstruction algorithms, attenuation correction (AC), and scatter correction but also to automate the segmentation of reconstructed images, increasing their accuracy and standardization.

### Reconstruction algorithm

The most used reconstruction algorithm in nuclear medicine is the ordered subset expectation maximization (OSEM) that consistently underestimates the standardized uptake value (SUV) [30]. New digital PET reconstruction algorithms, such as maximum likelihood algorithms, better control the reconstruction quality, however at the expense of vendor-specific software requirements and computational power [31, 32]. Hence, there is a need for AI techniques in this field [33].

Among different applications, the implementation of AI algorithms in PET/magnetic resonance imaging (MRI) reconstruction seems particularly interesting in the development of hybrid imaging in PCa patients. AC in PET/MRI is more challenging than in PET/CT, as voxel intensity of MRI cannot reflect photon attenuation characteristics directly; MRI-based AC (MRAC) methods mainly include segmentation-based methods (standard of care nowadays), atlas-based methods and template-based methods, which are easily affected by individual differences in anatomy, and emission and transmission-based methods, which are time-consuming. Researchers have developed several ML-based methods to improve the segmentation-based method of MRAC with AI, training mapping relationships to predict pseudo-CT (pCT) attenuation map from MRI data [34]. Different DL approaches have been developed to improve the MRAC segmentation-based method in the pelvic area through 3D deep CNN techniques [35–37], ultra-short echo time (UTE [38]) or Dixon volumetric interpolated breath-hold examination (Dixon-VIBE [39]) MRI sequences, and generative adversarial networks (GANs [40, 41]), as summarized in Table 1.

**Table 1:** Overview of retrospective studies on ML-based improvement of the segmentation-based MRAC method by AI

Author and publication year	Algorithm	Cohort (patients)	Ground truth	Performance
Bradshaw et al. (2018 [35])	DL-based attenuation-correction method (deepMRAC) = 3D-CNNs, namely DeepMedic (https://biomedia.doc.ic.ac.uk/soft-ware/deepmedic/) The network was trained to produce a discretized (air, water, fat, and bone) substitute computed tomography (CT) (CTsub). Discretized (CTref discrete) and continuously valued (CTref) reference CT images were created to serve as ground truth for network training and attenuation correction, respectively	Eighteen female patients with cervical cancer were randomly split into 12 training subjects and 6 testing subjects with [^18^F]FDG PET/MRI scan (with T2 MRI, T1 LAVA Flex, and 2-point-Dixon-based MRAC images) and following a PET/CT scan No validation cohort	Reference CT (CTref) images were generated by using a combination of different techniques for different tissue types. Bone = CT image + T2 MRI image followed by segmentation of the bone. Fat and water = fat-fraction image, generated from the 2-point Dixon acquisition. Air (including bowel gas) = intensity threshold of the T2 image on the basis of an ROI in the muscle, with manual corrections	The Dice coefficient of the AI-produced CTsub compared with CTref discrete was 0.79 for cortical bone, 0.98 for soft tissue, and 0.49 for bowel gas. The root-mean-square error (RMSE) of the whole PET image was 4.9% by using deepMRAC and 11.6% by using the system MRAC
Leynes et al. (2018 [36])	DL-based attenuation-correction method = U-net-CNNs, composed of 13 layers in total The deep learning model allowed a direct and fully automated conversion of MRI images to synthetic CT images, a so-called zero echo-time and Dixon Deep pseudoCT (“ZeDD-CT”), for PET image reconstruction (providing a patient-specific continuous-valued attenuation coefficients in soft tissues and in bone, respectively) and to evaluate the impact on radiotracer uptake estimation	Twenty-six patients with pelvic lesions (split into 10 training subjects and 16 evaluation subjects) and a PET/MRI scan performed with [^18^F]FDG or [^68^Ga]Ga-PSMA-11 PET/MRI No validation cohort	Helical CT images of the patients were acquired and were co-registered to the MRI images	Thirty bone and 60 soft tissue lesions were evaluated, and the SUVmax was measured. Comparing the MRAC methods with the ground-truth CTAC, there was a reduction factor of 4 of RMSE in PET quantification for bone lesions, of 1.5 of RMSE for soft tissue lesions
Mostafapour et al. (2021 [37])	DL-based attenuation-correction method = residual deep learning model, taking PET non-attenuation-corrected images (PET-NAC) as input and CT-based attenuation-corrected PET images (PET-CTAC) as target (reference)	Three-hundred ninety-nine whole-body [^68^Ga]Ga-PSMA-11 images were used as the training dataset Forty-six whole-body [^68^Ga]Ga-PSMA-11 images were used as an independent validation dataset	CT from corresponding PET-CTAC was used as reference (ground truth)	The AI method achieved a mean absolute error (MAE), relative error (RE%), structural similarity index (SSIM), and peak signal-to-noise ratio of 0.91 ± 0.29 (SUV), -2.46% ± 10.10%, 0.973 ± 0.034, and 48.171 ± 2.964, respectively, within images of the independent external validation dataset
Jang et al. (2018 [38])	DL-based attenuation-correction method = DL network via convolutional neural networks, which was pre-trained with T1-weighted MRI images. Ultrashort echo time (UTE) images are used as input to the network, which was trained using labels derived from co-registered CT images	Head PET/MRI of 8 human subjects No validation cohort	A registered CT image was used as ground truth	Dice coefficients for air (within the head), soft tissue, and bone labels were 0.76 ± 0.03, 0.96 ± 0.006, and 0.88 ± 0.01. In PET quantitation, the proposed MRAC method produced relative PET errors of less than 1% within most brain regions
Torrado-Carvajal et al. (2020 [39])	DL-based attenuation-correction method = Dixon-VIBE Deep Learning (DIVIDE). A deep-learning network that allows synthesizing pelvis pseudo-CT maps based only on the standard Dixon volumetric interpolated breath-hold examination (Dixon-VIBE) images	Twenty-eight datasets obtained from 19 patients who underwent PET/CT and PET/MRI examinations were used to evaluate the proposed method No validation cohort	CT from PET/CT	Absolute mean relative change values relative to CT AC were lower than 2% on average for the DIVIDE method in every ROI except for bone tissue, where it was lower than 4%
Pozaruk et al. (2021 [41])	DL-based attenuation-correction method = an augmented generative adversarial network (GAN) Aim to improve the accuracy of estimated attenuation maps from MRI Dixon contrast images by training an augmented generative adversarial network (GANs) in a supervised manner	Twenty-eight prostate cancer patients. Eighteen patients (2,160 slices and later augmented to 270,000 slices) were used for training the GANs, the remaining 10 patients for validation	CT images	The DL-based MRI methods generated the pseudo-CT AC μ-maps with an accuracy of 4.5% more than standard MRI-based techniques, thanks to the augmentation of the training datasets for the training of the GAN results in improved accuracy of the estimated μ-map and consequently the PET quantification compared to the state of the art

In single-photon emission tomography (SPECT)/CT image reconstruction, Ryden et al. [42] successfully evaluated a U-shaped convolutional deep neural network for the generation of synthetic intermediate projections (CUSIPs) to decrease the [^**177**^Lu]SPECT acquisition time by reducing the number of projections and to circumvent image degradation. The ML-based method studied on 352 SPECTs for training, 37 for validation and 15 for testing, appears to significantly recover image quality and allows reduced SPECT acquisition time in clinical dosimetry protocols. These results are of particular interest in the RLT approach for PCa.

### Segmentation process

Another promising use of ML is the automatic segmentation of lesions through a fully automated definition of regions of interest (ROI), mainly with the use of fully convolutional network (FCN) algorithms, such as the U-Net. Automatic segmentation could overcome the major problem of interobserver and intra-observer variability of manual segmentation, which is also time-consuming. This might increase the homogeneity and reproducibility of data, also for radiomics assessment [43, 44].

In 2019, Zhao et al. [45] developed a modified 2.5D U-Net architecture for the automatic segmentation of metastatic lesions in the pelvis extracted from [^**68**^Ga]Ga-PSMA-11 PET images of 71 patients. The preliminary test showed high accuracy in detecting bone lesions (recall = 0.98, precision = 0.97, F1 score = 0.98) and pathologic lymph nodes (recall = 0.84, precision = 0.75, F1 score = 0.79) but lower accuracy in detecting intraprostatic lesions (recall = 0.63, precision = 0.88, F1 score = 0.73). This promising CNN requires future implementation and validation in total-body assessment.

In 2021, Kostyszyn et al. [46] conducted a study on primary tumor delineation, developing a CNN (3D U-Net) network for automatic segmentation of intraprostatic gross tumor volume (GTV) in [^**68**^Ga]Ga-PSMA-11 PET. The network was trained on [^**68**^Ga]Ga-PSMA-11 PET images of 152 patients (training labels were manually generated), finding good agreement between fully automated segmentation and manual expert contouring on an external validation cohort and obtaining a median Dice similarity coefficient (DSC) of 0.81.

An interesting initiative is the Research Consortium for Medical Image Analysis (RECOMIA), an online platform developed by a nonprofit organization to facilitate collaborations between medical researchers and AI [47]. The platform has already been successfully used for detection and segmentation of primary prostate cancer [48, 49], bone metastases [50, 51], and lymph node metastases [52].

Concerning the primary tumor, Mortensen et al. [48] evaluated a CNN system in [^**18**^F] choline PET scans of 45 PCa patients before radical prostatectomy. Corresponding measurements were performed and compared with the weighted surgically removed tissue specimens and manually derived data. Assuming that 1 g equals 1 mL of tissue, the mean weight of the prostate specimens was 44 g, while CNN-estimated volume was 62 mL, with a mean difference of 13.5 g or mL (95% CI: 9.78–17.32). Moreover, automated CNN segmentation provided similar results to manually derived ones in terms of volume and conventional PET parameters. Polymeri et al. [49] sought to validate a DL algorithm for automated PCa quantification on [^**18**^F]choline PET images from 145 PCa patients (100 for testing and 45 for validation) and subsequently explored the potential of PET/CT measurements as prognostic biomarkers. The Sørensen-Dice index (SDI, a statistic index used to gauge the similarity of two samples) between automated and manual volume segmentations was 0.78 and 0.79, respectively. Moreover, automated PET/CT measurements were significantly correlated with overall survival (OS; *p* = 0.02), while age, prostate-specific antigen, and Gleason score were not.

Belal et al. [50] aimed to evaluate a 3D index for automatic segmentation of bone metastases on [^**18**^F]NaF PET/CT images and assessed its correlation with bone scan index (BSI) and OS in 48 PCa patients. Hotspots in PET images were selected either manually or automatically (using an SUV threshold > 15, PET15 index). BSI, manual PET, and automated PET15 index were all significantly correlated with OS, and concordance indices were 0.68, 0.69, and 0.70, respectively. A second study from the same group [51] aimed to develop a DL-based method for bone segmentation in CT scans and test its accuracy compared with manual delineation to quantify skeletal tumor burden by testing it on 46 PCa patients, who underwent [^**18**^F]choline and [^**18**^F]NaF PET/CT within 3 weeks. The network performance was compared with manual segmentations of five skeletal districts segmented manually by an experienced physician; median SDIs were 0.86 for Th7, 0.85 for L3, 0.88 for the sacrum, 0.84 for 7th rib, and 0.83 for the sternum. The intra-observer volume difference was smaller with the CNN-based approach than with the manual approach.

Borrelli et al. [52] tested two CNN networks for the automatic segmentation of [^**18**^F]choline PET/CT images in 399 PCa patients (319 for training and 80 for testing); one neural network detected the target organs for prostate disease on CT images, while the other one used this result together with the PET image to automatically detect lymph node metastases. Results were compared with those of two independent expert readers; the AI-based instrument detected more lymph node lesions than one reader (98 *versus* 87/117; *p* = 0.045) using the other reader as reference, while the AI performed similar to the other reader (90 *versus* 87/111; *p* = 0.63), using the first reader as reference. In addition, the number of automatically detected lymph node metastases was significantly associated with PCa-specific survival (HR = 1.19, 95% CI 1.05–1.33).

## Clinical applications

Nuclear medicine plays a predominant role in the noninvasive assessment of PCa in terms of staging, treatment response assessment, and RLT eligibility assessment, all of which can be improved by radiomics and AI.

### Staging

Several authors have attempted to improve the accuracy of nuclear medicine examinations in the staging of PCa patients by applying radiomics, ML, and DL approaches.

In 2019, Zamboglou et al. [53] extracted RFs from [^**68**^Ga]Ga-PSMA-11 PET/CT of two cohorts of intermediate/high-risk PCa patients, one prospective (20 patients) and one retrospective (40 patients) cohort, who afterwards underwent radical prostatectomy and pelvic lymph node dissection. RFs extracted from the manual segmentations (GTV-Exp) showed strong correlations with RFs extracted from co-registered histopathological gross tumor volume (GTV-Histo = ground truth; 86% with *p* > 0.7), both discriminating significantly between PCa and non-PCa tissue. The texture feature QSZHGE discriminated between GS 7 and ≥ 8 for GTV-Exp (prospective cohort AUC = 0.91, validation cohort AUC = 0.84) and also between nodal spread (pN1) and non-nodal spread for GTV-Exp (prospective cohort AUC = 0.87, validation cohort AUC = 0.85). In the multivariate analyses, QSZHGE was a significant predictor (*p* < 0.01) for PCa patients with GS ≥ 8 tumors and pN1 status.

In 2020, Cuzzocrea et al. [54] performed a radiomics study using 42 high-risk PCa patients staged with [^**18**^F]choline PET to assess the relationship between texture analysis of prostatic [^**18**^F]choline uptake and patient outcome. For each patient, they calculated the RFs, metabolic parameters of the prostate gland, and the risk assessment score (RAS, based on PSA levels, Gleason score, and T classification). Among 38 RFs, 19 were statistically different between patients with stable disease and patients with biochemical progression at follow-up (*p* < 0.03). GLCM contrast (Se = 77.8; Sp = 84.8; PPV = 58.3; NPV = 93.3; cutoff = 9.9) and GLZLM-HGZE (Se = 77.8; Sp = 87.9; PPV = 63.6; NPV = 93.5; cutoff = 151.4) showed the best performance for predicting patient outcome (median follow-up 19.8 months), with AUCs of 0.828 and 0.858 (both *p* < 0.001), respectively.

In 2021, Zamboglou et al. [55] investigated two cohorts of primary PCa patients, a prospective training cohort (*n* = 20), and an external validation cohort (*n* = 52). They aimed to find PSMA-PET-derived RFs able to detect intraprostatic lesions missed by visual [^**68**^Ga]Ga-PSMA-11 PET/CT assessment. Visual PSMA-PET image interpretation missed 134 PCa lesions (median of 2 missed lesions per patient) with a median maximum diameter of 4 mm (range: 2–6). PCa was missed in 60% of patients in the training cohort (75% with clinically significant PCa, ISUP > 1) and in 50% of patients in the validation cohort (77% with clinically significant PCa). Local binary pattern (LBP) normalized size-zone non-uniformity and LBP small-area emphasis were the only two RFs capable of identifying occult PCa (*p* < 0.01), with an AUC ≥ 0.93 in the training cohort and AUC ≥ 0.80 in the validation cohort.

In 2015, Gatidis et al. [56] performed the first ML-based radiomic study on 16 PCa patients who underwent staging [^**18**^F]choline PET/MRI. A spatially constrained fuzzy c-means algorithm (sFCM) was applied to the single datasets, and the resulting labeled data were used for training a SVM classifier. Accuracy and false-positive/negative rates of the proposed algorithm were determined in comparison with manual tumor delineation or histopathology correlation in 5 of 16 patients. The combined sFCM/SVM algorithm revealed reliable classification results consistent with the histopathological reference standard and comparable to those of manual tumor delineation. Also, sFCM/SVM generally performed better than unsupervised sFCM alone.

In 2021, other authors evaluated the prognostic value of RFs extracted from nuclear medicine images through the help of ML-based techniques. Cysouw et al. [57] conducted a radiomics study on a cohort of 76 intermediate/high-risk PCa patients, who underwent [^**18**^F]DCFPyL PET/CT before radical prostatectomy. They aimed to develop a diagnostic ML-based model for detecting the presence of metastases (pelvic lymph node or distant metastases). RFs were chosen via three different feature selection methods: principal component analysis (PCA), recursive feature elimination with random forest, and univariate analysis of variance utilizing the fivefold cross-validation. The resulting random forest algorithm achieved a good discriminatory performance in the detection of lymph node or distant metastasis (both AUC = 0.86, *p* < 0.01), leading to a noninvasive determination of low-risk patients that could be spared from extended pelvic lymph node dissection.

Papp et al. [58] aimed to investigate the diagnostic performance of dual-tracer ([^**18**^F]choline and [^**68**^Ga]Ga-PSMA-11) PET/MRI in 52 PCa patients undergoing radical prostatectomy, to predict low-risk *versus* high-risk lesions (LH) as well as biochemical recurrence risk (BCR) and overall patient risk (OPR) with ML. RFs, extracted from both [^**68**^Ga]Ga-PSMA-11 PET and MRI images, in combination with ensemble ML, were applied and compared with conventional PET parameters. The AUC of the ML-based LH model was higher than the SUV_max_ analysis (0.86 *versus* 0.80); the accuracies of the BCR model and OPR model were 89% (AUC = 0.90) and 91% (AUC = 0.94), respectively.

Erle et al. [59] aimed to compare and validate supervised ML algorithms to classify pathological uptake in PCa patients based on [^**68**^Ga]Ga-PSMA-11 PET/CT images. Authors evaluated 77 RFs from 2452 manually delineated hotspots (1,629 pathological *versus* 823 physiological, as ground truth) for the training dataset (72 PCa patients) and 331 hotspots (pathological = 128, physiological = 203) for the validation dataset (15 PCa patients). Three ML classifiers were trained and ranked to assess classification performance. A high overall average performance (AUC = 0.98) was achieved, with higher sensitivity for the detection of pathological uptake (sensitivity = 0.97) compared with physiological uptake (sensitivity = 0.82).

The first DL-based study was conducted in 2020 by Hartenstein et al. [60]; they assessed if CNNs can be trained to determine [^**68**^Ga]Ga-PSMA-11 PET/CT lymph node status from CT images of 549 PCa patients, evaluating 2,616 lymph nodes identified on PET. The CNN for the binary classification of lymph nodes achieved an accuracy of 89% (AUC = 0.95; Sens = 86%; Spec = 92%) in the training group but failed in the external validation. Hence, this approach is not generalizable, and its value remains unclear.

Moreover, Capobianco et al. [61] developed a DL approach, investigating the use of training information from two radiotracers, [^**68**^Ga]Ga-PSMA-11 and [^**18**^F]FDG. With limited PSMA-ligand data available, the idea was that the use of training examples from [^**18**^F]FDG, a more widely used radiotracer in general oncology, should improve the performance of the DL approach for the assessment of [^**68**^Ga]Ga-PSMA-11 images. The CNN network was developed on a larger [^**18**^F]FDG PET/CT image dataset (of lymphoma and lung cancer patients), also assessing transfer learning and the ability to encode tracer type. Then, the developed CNN method was trained on [^**68**^Ga]Ga-PSMA-11 PET/CT of 173 patients, divided into development (121) and test (52) sets, to both classify sites of increased tracer uptake as non-suspicious/suspicious for cancer and assign an anatomical location. The expert annotations for the N and M status, according to the PROMISE miTNM framework, were used as ground truth. The evaluated algorithm showed good agreement with expert assessment for the identification and anatomical location classification of suspicious uptake in whole-body [^**68**^Ga]Ga-PSMA-11 PET/CT.

Finally, Solari et al. [62] evaluated the performance of combined [^**68**^Ga]Ga-PSMA-11 PET and mpMRI image biomarker standardization initiative (IBSI)-compliant RFs for the group-wise prediction of postsurgical GS (psGSs) in 101 primary PCa patients, divided into three categories (ISUP grades 1–3, ISUP grade 4, and ISUP grade 5). Nine SVM models were trained: four single-modality radiomics models (PET, T1w, T2w, ADC), three PET+MRI double-modality models, and two baseline models for comparison. A sixfold-stratified cross-validation was performed, and all radiomic models outperformed the baseline models. The overall best-performing model combined PET+ADC radiomics (82%). It significantly outperformed most of the others dual-modality models (PET + T1w: 74%, *p* = 0.026; PET + T2w: 71%, *p* = 0.003) and single-modality models, except the ADC-only model (*p* = 0.138).

### Restaging

In 2020, Kang et al. [63] developed a computational methodology using Haralick texture analysis that can be used as an adjunct tool to improve and standardize the interpretation of FACBC PET/CT images to identify BCR, discerning necrotic tissue from radiation therapy and tumor tissue in 28 PCa patients. Four main RFs were chosen and combined with clinical information; the overfitting-corrected AUC and Brier scores of the proposed model were 0.94 (95% *CI*: 0.81, 1.00) and 0.12 (95% *CI*: 0.03, 0.23), respectively.

Other authors evaluated different ML-based approaches with different aims. In 2020, Lee et al. [64] examined with an ML-based approach the [^**18**^F]fluciclovine PET images of a cohort of 251 PCa patients with suspected BCR following definitive primary therapy, to automatically identify “normal” patients (no disease recurrence) and “abnormal” patients (locoregional or distant recurrence). CNN models were trained using two different architectures, a 2D-CNN (ResNet-50), using single slices (slice-based approach), and the same 2D-CNN with a 3D-CNN (ResNet-14), using a hundred slices per PET image (case-based approach). The best prediction results were achieved by the 2D slice-based CNN (AUC = 0.971, *p* < 0.001; Sens = 90.7%; Spec = 95.1%). The underperformance of 3D-CNN compared to 2D-CNN could derive from a larger number of learnable parameters in 3D-CNN and would therefore require a larger training dataset size to generate a sufficiently generalizable model.

Moazemi et al. [65] employed five different ML methods on RFs (40 from PET images and 40 from CT images) to classify 2419 [^**68**^Ga]Ga-PSMA-11 PET hotspots in 72 patients (48/72 applied for training) as either benign or malignant. Interestingly, RFs assessed in native low-dose CT increased the accuracy significantly. The ML method achieved better accuracy (AUC = 0.98; Sens = 94%, Spec = 89%) than human readers.

Alongi et al. [66, 67] evaluated the potential application of RFs analysis using an ML-based radiomic algorithm to select [^**18**^F]choline PET/CT features to predict disease progression in high-risk BCR PCa patients. In their study [67], the authors analyzed 94 high-risk PCa patients who underwent [^**18**^F]choline PET/CT restaging imaging to select features able to predict disease progression (median follow-up of 26 months). Discriminant analysis on the RFs extracted yielded an ML model capable of achieving moderate predictive power in the development of nodal (AUC = 69.87, 95% *CI* 51.34–88.39) or distant metastases (AUC = 74.72, 95% *CI* 56.36–93.09). HISTO_entropy_log10 and HISTO_entropy_log2 were the two salient features chosen for the discrimination of distant metastases, while GLSZM_SZLGE and HISTO_energy_uniformity were the chosen features to predict nodal metastases.

### Bone metastasis

Bone scintigraphy is a reference standard examination to assess bone metastatic spread of PCa patients. In 2021, Cheng et al. [68] aimed to explore efficient ways to early diagnose bone metastasis using bone scintigraphy images through ML methods in two cohorts of 205 PCa patients and 371 breast cancer patients. Authors used bone scintigraphy data from breast cancer patients to pre-train a YOLO v4 with a false-positive reduction strategy and then trained the approach on a dataset of 194 PCa patients under a tenfold cross-validation scheme, which yielded a lesion-level classification sensitivity of 0.72 and a precision of 0.9.

Trying a DL approach, Ntakolia et al. [69] designed a DL method that overcomes the computational burden by using a CNN with a significantly lower number of floating-point operations (FLOPs) and free parameters comparing to other popular and well-known CNN architectures used for medical imaging, such as VGG16, ResNet50, GoogleNet, and MobileNet. The proposed lightweight look-behind fully CNN architecture was used to classify bone scintigraphy images of 778 metastatic PCa patients into three classes: no metastasis, degenerative (defined as the absence of metastasis but presence of degenerative lesions), and metastatic lesions. The final optimal CNN achieved a high accuracy of 91.6% (F1 score = 0.938). Furthermore, the best-performing CNN method was compared to the other abovementioned CNN architectures used for medical imaging, outperforming the others. These results were similar to previous results of the same group [70], using the same CNN architecture for bone scintigraphy images of 586 metastatic PCa patients divided into only two classes (no metastasis and metastasis), resulting in a higher overall accuracy of 97.38% than the one in the previous study.

In this field, also [^**18**^F]NaF PET/CT might be useful, having a higher accuracy than bone scan [17, 71]. In 2018, Perk et al. [72] delineated the [^**18**^F]NaF PET/CT images of 37 mCRPC patients by an automated algorithm that determines the lesion boundaries based on statistically optimized regional thresholding (SORT). A classification labeled by an expert depending on the likelihood of malignancy (from 0 = background, 1 = definitely benign to 5 = definitely malignant) was applied to 123 bone lesions. Furthermore, the RFs extracted have been used in the ML analysis with nine separate learning methods, where the random forest model performed the best under tenfold cross-validation conditions at discriminating between the 0 + 1 *versus* 5 class labels (AUC = 0.95, 95% CI 0.93–0.96).

Albeit with a non-bone-specific tracer, Acar et al. [73] using RFs aimed to distinguish lesions imaged via posttreatment [^**68**^Ga]Ga-PSMA-11 PET/CT as nonresponding and completely responding (sclerotic lesions) in 75 PCa patients with known bone metastasis. Sclerotic lesions were categorized as complete responding or nonresponding if they showed [^**68**^Ga]Ga-PSMA-11 PET uptake levels either below or above liver uptake, respectively. Multiple ML models were developed, and the weighted K-nearest neighbor (KNN) achieved the best classification performance under tenfold cross-validation conditions with AUC = 0.76 (accuracy = 73.5%, sensitivity = 73.5%, specificity = 73.7%).

Finally, in 2021, Hinzpeter et al. [74] investigated the potential application of RFs analysis using an ML-based radiomics algorithm for detecting bone metastases not visible on low-dose CT, extracting from [^**68**^Ga]Ga-PSMA-11 PET imaging of 67 patients with PCa as the reference standard (ground truth). The authors analyzed a total of 205 bone metastases with PSMA avidity, but not visible on low-dose CT. The dataset was divided into training, testing, and validation, which allowed the selection of 11 independent RFs. A gradient-boosted tree was trained on the 11 RFs to classify bones as normal or metastatic, using the training dataset. The model achieved a classification accuracy of 0.85 (95% confidence interval [CI]: 0.76–0.92, *p* < .001) with 78% sensitivity and 93% specificity.

### Theragnostics

PSMA-RLT is an emerging treatment modality for advanced PCa [18]. However, almost 30% of patients do not respond to [^**177**^Lu]PSMA RLT, which may be due to intralesional and inter-lesional variations of PSMA expression, potentially resulting in undertreatment and reduced RLT efficacy. The early identification of patients who might benefit from RLT can be supported by pre-therapeutic biomarkers derived from radiomics and AI analysis.

In 2018, Khurshid et al. [75] aimed to assess the predictive ability of tumor textural heterogeneity parameters in a total of 328 metastatic lesions from baseline [^**68**^Ga]Ga-PSMA-11 PET/CT of 70 mCRPC patients scheduled to undergo [^**177**^Lu]PSMA therapy. NGLCM_Entropy showed a negative correlation (*rs* = -0.327, *p* = 0.006, *AUC* = 0.695), and NGLCM_Homogeneity showed a positive correlation (*rs* = 0.315, *p* = 0.008, *AUC* = 0.683) with pre- and post-therapy PSA levels, where a reduction in PSA classified patients as responders (42/70) and an increase in PSA as nonresponders (28/70).

More recently, Moazemi et al. [76] extracted RFs from 2070 malignant hotspots from 83 advanced PCa patients delineated at pre-therapeutic [^**68**^Ga]Ga-PSMA-11 PET/CT scan to analyze the OS of patients treated with RLT. Following a LASSO regression feature selection process, the most relevant RFs (PET kurtosis and SUV_min_) significantly correlated with OS (*r* = 0.2765, *p* = 0.0114).

In 2021, Roll et al. [77] evaluated the predictive and prognostic value of RFs extracted from [^**68**^Ga]Ga-PSMA-11 PET/MRI in 21 mCRPC patients before RLT. The PET-positive tumor volume was defined and transferred to whole-body T2-weighted and contrast-enhanced and non-enhanced T1-weighted MRI pulse sequences. Ten independent RFs differentiated well between responders (8/21) and nonresponders’ patients (13/21), and the logistic regression model, including the feature interquartile range fromT2-weighted images, revealed the highest accuracy (AUC = 0.83) for the prediction of biochemical response after RLT. Within the final model, patients with a biochemical response (*p* = 0.003) and higher T2 interquartile range values in pre-therapeutic imaging (*p* = 0.038) survived significantly longer.

Finally, Götz et al. [78] investigated how to introduce a dosimetry method where dose voxel kernels (DVK) are predicted by a neural network based on data acquired of the kidneys in 26 patients undergoing therapy with [^**177**^Lu]PSMA or [^**177**^Lu]DOTATOC, as target organs of the experimental dosimetric method. The method, implemented on SPECT/CT images, was found accurate and competitive when compared to the standard, in which the activity distribution is convolved with a DVK based on a homogeneous soft-tissue kernel.

## Discussion

This review highlights all possible uses of radiomics and AI in the clinical PCa scenario. The use of AI in PET/MRI image reconstruction could overcome the problems related to MRAC methods currently in use in clinical practice, reducing errors related to individual differences in anatomy and reducing image reconstruction time.

Another extremely interesting application is the automatic segmentation of the tumor, with very useful implications in clinical practice; an automatic segmentation of the primary lesion could implement fusion biopsy systems using simultaneously ultrasound, MRI, and PET data to identify more accurately the target site for biopsy, while an automatic segmentation of metastases, particularly skeletal metastases, would allow accurate and reproducible assessment of tumor burden and response to systemic treatment.

There are several possible clinical applications in PCa staging; radiomics and AI can help to discriminate healthy from pathological prostate tissue, to correlate prostate lesion with GS, ISUP grade, and N status, or to predict low-risk *versus* high-risk lesions, as well as biochemical recurrence risk and overall patient risk. While in PCa restaging, radiomics and AI can improve the interpretation of PET/CT images to identify BCR by discerning post-radiotherapy necrotic tissue from tumor tissue, to automatically recognize patients without disease recurrence from patients with locoregional-distant recurrence, to automatically classify bone lesions as degenerative or metastatic lesions, but also to predict disease progression in PCa patients at high BCR risk. Finally, radiomics and AI are also useful tools to better identify responder and non-responder patients in the therapeutic setting.

Despite this enormous potential of radiomic and AI in nuclear medicine PCa field, their application in clinical practice is still challenging and not yet feasible; myriad of factors can affect the resulting quantitative imaging biomarker measurement; imaging analysis procedures such as tumor segmentation methods, gray-level intensity discretization, and image reconstruction algorithm can affect robustness, repeatability, and reproducibility of these variables and their results [22, 79–83].

Recently, several documents have been provided by the scientific community to increase the robustness of these tools, such as the radiomics quality score (RQS) [23], a point-based system that guides the researcher to use a rigorous methodological approach for performing radiomics, and the imaging biomarker standardization initiative (IBSI) [84] that aim to provide image biomarker nomenclature and definitions, benchmark data sets, and benchmark values to verify image processing and image biomarker calculations, as well as reporting guidelines, for high-throughput image analysis. Finally, in a recent interesting review, Zwanenburg [81] identified and described the main pitfalls of data analysis that affect the reproducibility and generalizability of radiomics studies, dividing them into macro-areas: patient selection (sample size, injected radiopharmaceutical activity, patient movement, etc.), image acquisition (characteristics of the tomograph and type of acquisition used), image reconstruction (number of iterations, subsets, etc.), segmentation, image processing, image biomarker computation, and modeling.

These instruments are increasing researchers’ understanding of the more technical aspects of radiomic and AI studies, leading to a gradual harmonization and standardization of these approaches and making the radiomic and AI possible future application in clinical settings more than just a hypothetical mirage.

## Conclusion

Radiomics and AI approaches are receiving increasing attention from the scientific community due to several potential applications in PCa patients: from the technical aspects of image reconstruction and segmentation, which also allow optimization of workflow, to clinical aspects, such as lesion classification and image evaluation in terms of predictiveness and prognosis. However, a considerable workload and several validation studies are still needed to introduce most of these methods in clinical practice. In fact, most of these approaches are currently limited by the need to collect a large number of data, which is in conflict with the growing concern about privacy. Nevertheless, for personalized medicine, AI applications will be essential to manage and integrate the large amounts of quantitative data from medical images with clinical data. In this sense, the use of AI in state-of-the-art simultaneous PET/MRI is desirable, potentially enhancing molecular imaging applications in precision medicine.

## Data Availability

Not applicable

## Abbreviations

[^18^F]FDG: 2-deoxy-2-[^18^F]fluoro-D-glucose
AC: Attenuation correction
AI: Artificial intelligence
BCR: Biochemical recurrence
CNNs: Convolutional neural networks
DL: Deep learning
FACBC: [^18^F]flurocyclobutane-1-carboxylic acid
GANs: Generative adversarial networks
GTV: Gross tumor volume
mCRPC: Dedifferentiated, metastatic, castration-resistant PCa
ML: Machine learning
MRAC: MRI-based AC
OS: Overall survival
PCa: Prostate cancer
PET: Positron emission tomography
PSMA: Prostate-specific membrane antigen
RFs: Radiomic features
RLT: Radioligand therapy
ROI: Regions of interest
sFCM: Spatially constrained fuzzy c-means algorithm
SPECT: Single-photon emission tomography
SUV: Standardized uptake value
SVM: Support vector machine
